# Accuracy and safety of C2 pedicle or pars screw placement: a systematic review and meta-analysis

**DOI:** 10.1186/s13018-020-01798-0

**Published:** 2020-07-20

**Authors:** Parisa Azimi, Taravat Yazdanian, Edward C. Benzel, Hossein Nayeb Aghaei, Shirzad Azhari, Sohrab Sadeghi, Ali Montazeri

**Affiliations:** 1 Department of Neurosurgery, Shahid Beheshti University of Medical Sciences, Arabi Ave, Daneshjoo Blvd, Velenjak, Tehran, 19839-63113 Iran; 2 School of Medicine, Capital Medical University, Beijing, China; 3 Department of Neurosurgery, Cleveland Clinic Foundation, Cleveland, OH USA; 4 Population Health Research Group, Mental Health Research Group, Health Metrics Research Centre, Iranian Institute for Health Sciences Research, ACECR, Tehran, Iran

**Keywords:** Upper cervical, Fusion, C2 pedicle, C2 pars, Radiographic malposition, Accuracy rate, Free-hand, Navigation

## Abstract

**Study design:**

Systematic review and meta-analysis.

**Aim:**

The purpose of this study was to compare the safety and accuracy of the C2 pedicle versus C2 pars screws placement and free-hand technique versus navigation for upper cervical fusion patients.

**Methods:**

Databases searched included PubMed, Scopus, Web of Science, and Cochrane Library to identify all papers published up to April 2020 that have evaluated C2 pedicle/pars screws placement accuracy. Two authors individually screened the literature according to the inclusion and exclusion criteria. The accuracy rates associated with C2 pedicle/pars were extracted. The pooled accuracy rate estimated was performed by the CMA software. A funnel plot based on accuracy rate estimate was used to evaluate publication bias.

**Results:**

From 1123 potentially relevant studies, 142 full-text publications were screened. We analyzed data from 79 studies involving 4431 patients with 6026 C2 pedicle or pars screw placement. We used the Newcastle-Ottawa Scale (NOS) to evaluate the quality of studies included in this review. Overall, funnel plot and Begg’s test did not indicate obvious publication bias. The pooled analysis reveals that the accuracy rates were 93.8% for C2 pedicle screw free-hand, 93.7% for pars screw free-hand, 92.2% for navigated C2 pedicle screw, and 86.2% for navigated C2 pars screw (all, *P* value < 0.001). No statistically significant differences were observed between the accuracy of placement C2 pedicle versus C2 pars screws with the free-hand technique and the free-hand C2 pedicle group versus the navigated C2 pedicle group (all, *P* value > 0.05).

**Conclusion:**

Overall, there was no difference in the safety and accuracy between the free-hand and navigated techniques. Further well-conducted studies with detailed stratification are needed to complement our findings.

## Background

Atlantoaxial instability or upper cervical spine instability is defined as excessive mobility as a result of either a bony or ligamentous abnormality [1]. Operative treatment of atlantoaxial instability is performed with a variety of fixation techniques. Spinous process wiring techniques were developed in 1910; laminar wiring techniques were developed in 1939; C1–2 laminar and modified posterior wiring technique were developed in 1991 [2]. These techniques did not provide sufficient biomechanical stability [2]. To address this matter, the C1–C2 transarticular screw fixation technique was introduced in 1992 [3]. However, 22% of cases were not appropriate candidates for transarticular screws because of an increased risk of vertebral artery injury [4]. Some more recently developed methods of C1–C2 fixation, C1 lateral mass screws combined with C2 pedicle/pars/laminar screws, have enhanced the stability of the upper cervical spine fixation techniques [2, 5]. C2 pedicle screw placement was first described by Goel et al. in the 1980s [2].

An alternative to the prior mentioned techniques is the pars screw, sometimes referred to as an isthmus screw. C2 screw fixation techniques have been enhanced by the development of poly-axial screws and top-loading rods [2]. Researchers showed that C2 pars and pedicle screw utilization leads to high rates of arthrodesis [5, 6]. These techniques are also employed in the subaxial cervical spine [5]. C2 pedicle and pars screws require accurate placement to avoid injury to vital structures, such as the vertebral artery, spinal cord, and nerve roots [2, 5].

Overall, navigated and free-hand technique has been reported in detail elsewhere [7]. CT-based intraoperative navigation can be applied to determine a safe trajectory for C2 pedicle and pars screws placement but may be associated with increased time for image acquisition, increased radiation exposure to the patient, and possible registration inaccuracies. On the other hand, the free-hand technique minimizes radiation exposure to the surgeon and patient [5].

No systematic reviews to date have compared the accuracy and safety of C2 pedicle and pars screws placed with the free-hand technique to the safety and accuracy of screws placed with the assistance of navigation. Therefore, the purposes of this systematic review and meta-analysis are (1) to assess C2 pedicle and pars screw placement accuracy and (2) to evaluate the difference in C2 pedicle and pars screw placement accuracy between free-hand and navigation techniques based on radiographic malposition.

## Methods

### Search strategy

The research strategy was designed around the PICO (Patient, Intervention, Comparison, and Outcome) question format. The present review was performed, based on the Preferred Reporting Items for Systematic Reviews and Meta-analyses (PRISMA) guidelines [8]. Electronic searches were performed using the Scopus, PubMed, Web of Science, and Cochrane Library databases up to April 2020. The literature involving all comparative studies were searched, containing the following search terms: “C2 pedicle,” “C2 pars,” “atlantoaxial instability,” “upper cervical,” “spine,” “CT-based technique,” “navigated technique,” “craniocervical,” “freehand technique,” “screws,” “screws placement,” “accuracy rate,” and “safety.”

### Inclusion and exclusion criteria

All identified articles were systematically evaluated against the inclusion and exclusion criteria, independently reviewed by 2 authors, and disagreements were sent to third author for resolution. Any disagreement was resolved by discussion to reach a consensus. The inclusion criteria were as follows: studies presented accuracy rate in pedicle and/or pars C2 screw placement, based on either the free-hand or navigation techniques.

In recent years, different navigation systems such as fluoroscopic navigation, MR-based navigation [9], CT-guided navigation, and O-arm–based navigation have been developed for pedicle/pars screw placement guidance. In this study, all of these techniques were considered navigation systems. The free-hand technique is defined by the placement of C2 pedicle or pars screws without the use of any of the aforementioned navigation systems [7]. In addition, screw guide templates and accuracy of preoperative imaging in predicting of trajectory and size of screw were considered free-hand technique.

The exclusion criteria were as follows: (I) duplicate publications; (II) reviews, case reports, commentary, and letters; (III) studies not published in English; (IV) studies which C2 screw sample size < 15; and (V) studies without available data regarding statistical techniques and lack of radiographic malposition reporting; (VI) studies with anterior cervical surgery; (VII) studies regarding cadavers; (VIII) anatomical and biomechanical studies; (IX) studies regarding without detailed information of C2; and (X) studies without separate C2 pedicle and pars screw placement information.

### Data extraction

Two authors independently extracted the data from all eligible studies. The following data was extracted using a structured data extraction form from full articles: the first author, year of publication, country, sample size, gender, age, number of patients in C2 pars group in free-hand and navigation approach, number of patients in C2 pedicle group in free-hand and navigation approach, accuracy classification for assessing C2 pedicle/pars screw placement, and accuracy rate in four subgroups as pedicle, pars free-hand and pedicle, and pars navigation technique based on radiological malposition.

### Quality assessment

Identified studies were exported to Endnote version 7, and duplicates were removed. Two independent reviewers performed a full-text quality review. Disagreement between the two reviewers was resolved via discussion and a third author if needed. The NOS [10] was applied to evaluate the quality and risk of bias in included studies. The NOS includes 3 categorical criteria with a maximum score of 9 points: “selection” which accounts a maximum of 4 points, “comparability” which accounts a maximum of 2 points, and “outcome” which accounts a maximum of 3 points. No studies were randomized controlled trials; hence, studies with 7–9 points could be identified as high quality, 5–6 points as moderate quality, and 0–4 as poor quality. A summary of the procedure of quality assessment is presented in Table 1.

**Table 1:** Check list for quality assessment and scoring of studies based on NOS

Check list	
*Selection*	
1. Representativeness of the sample. Truly representative or somewhat representative? (if yes, one star)	
2. Sample size ≥ 40 (if yes, one star)	
3. How representative was the C2 pedicle group in comparison with C2 pars screw placement in upper cervical patients, and the accuracy rate assessment is satisfactory? (if yes, one star; no star if the patients were selected only in one group)	
4. Ascertainment of the risk factors as surgical record: Were the risk factors measured with valid and reliable instruments? (if yes, one star)	
*Comparability*	
The accuracy rate screw placement and any additional factors as age, gender, and accurate classification of radiological malposition in different outcome groups are comparable, based on the study design or analysis. Confounding factors are controlled. (if yes, two stars; one star was assigned if one any additional factors was not reported)	
*Outcome assessment*	
6. Ascertainment of the outcome: clearly defined outcome of accuracy rate (yes, two star for information ascertained by record accuracy rate based on classification of radiological malposition; one star if this information was not reported)	
7. Appropriate statistical analysis: The statistical test used to analyze the accuracy rate is clearly described and appropriate for C2 pedicle or pars pedicle (if yes, one star; no star was assigned if the accuracy rate is reported overall)	

### Statistical analysis

The raw data were entered into Microsoft Excel. Exact tests were calculated with SPSS. Only mean values were reported for the variables age at surgery and the number of patients; these variables were only semi quantitatively compared. In studies that did not report the age of C2 pedicle/pars screw group, the mean age was considered. In addition, in some of studies, the number of unreported cases was determined by dividing by two the number of the C2 pedicle/pars. Also, in some of studies, overall accuracy rates were considered for subgroups.

The meta-analysis was performed by using the Comprehensive Meta-Analysis version 2 (Biostat, Englewood, NJ). We assumed that the methodology of each study was unique, and the studies were heterogeneous. I-squared statistics were used to evaluate the heterogeneity of pooled accuracy rate estimates. If the I-squared value was > 50% and *P* value < 0.05, there was significant heterogeneity among the included studies, and a random effects model was applied to estimate the pooled results. Publication bias was estimated using Begg’s funnel plot. A 2-tailed *P* value of less than 0.05 was considered statistically significant for all analyses.

## Results

### Descriptive statistics

**Fig. 1:**
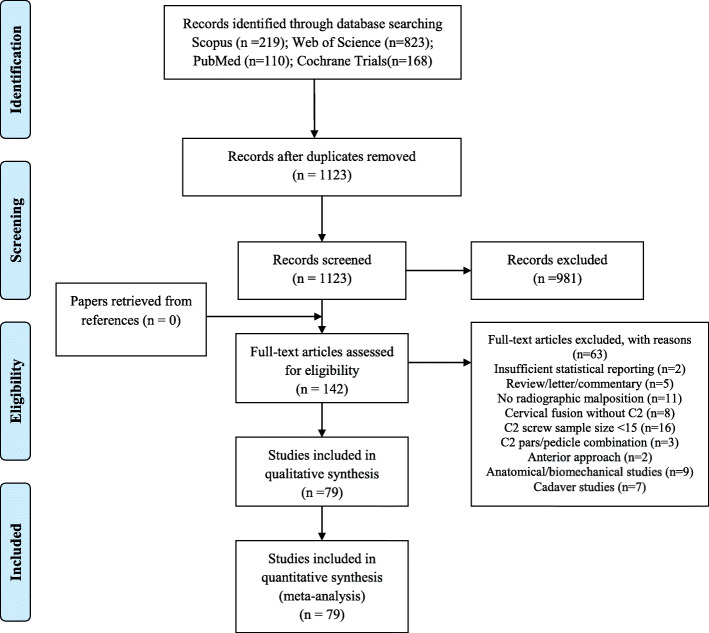
The results of the search strategy as performed by under the Preferred Reporting Items for Systematic Reviews and Meta-Analyses (PRISMA) guidelines

The literature search identified a total of 1320 articles. Figure 1 shows the flow diagram for the selection process for the systematic review. After removing 197 duplicated articles, 1123 remaining records were screened for title and abstract. Of those articles, 981 were excluded. Thus, 142 articles were assessed for eligibility by reading the full text. No randomized controlled trials were identified. Seventy-nine articles including 67 retrospective studies and 12 prospective studies were included for meta-analysis. The mean age of patients was 49.9 ± 13.3 years, and 57.4% of patients were male. A tabulated summary of the all studies are presented in Table 2 [5, 9, 11–87].

**Table 2:** Characteristics of included studies and quality assessment

Author(s) [Ref.]	Year	Country	Number of C2 screws used				Sample size (*n*)	Age mean (SD, range) years	Gender ratio (M:F)	Design	Assessing C2 screw placement accuracy classification	Accuracy rate (%)				Study quality
			Free-hand		Navigation							Free-hand		Navigation		
			Pedicle	Pars	Pedicle	Pars						Pedicle	Pars	Pedicle	Pars	
Abumi et al. [11]	2000	Japan	74	NR	NR	NR	74 out of 669 screw of 180 patients	70 (13–84) of 180 patients	106:74	Retrospective	Post-op CT, without classification	95.9 (71/74)	NR	NR	NR	6
Harms et al. [12]	2001	Germany	74	NR	NR	NR	37	49 (2–90)	19:18	Retrospective	Postoperative X-rays, without classification	100 (74/74)	NR	NR	NR	6
Goel et al. [13]	2002	India	320	NR	NR	NR	160	23 (1.7–79)	91:69	Retrospective	Satisfactory was considered, if the screw did not protrude more than 4 mm beyond the anterior cortex of the lateral mass of the atlas and axis	98.1 (314/320)	NR	NR	NR	8
Chen et al. [14]	2005	Taiwan	22	NR	NR	NR	11	48.6 (21–73)	8:3	Retrospective	Post-op CT, without classification	86.4 (19/22)	NR	NR	NR	5
Ondra et al. [15]	2006	USA	117	33	NR	NR	79	48 (15–91)	45:34	Retrospective	Post-op CT, without classification	91.4 (107/117)	96.9 (32/33)	NR	NR	7
Stulik et al. [16]	2007	Czech Republic	56	NR	NR	NR	28	59.5 (23–89)	18:10	Retrospective	Post-op CT, without classification	94.6 (53/56)	NR	NR	NR	6
Yeom et al. [17]	2008	South Korea	39	NR	NR	NR	23	47 (7–69)	15:8	Retrospective	Modified Gertzbein and Robbins	79.5 (31/39)	NR	NR	NR	7
Li et al. [18]	2008	China	42	NR	NR	NR	23	38 (19–52)	16:7	Retrospective	Postoperative X-rays, without classification	100 (42/42)	NR	NR	NR	6
Sciubba et al. [19]	2009	USA	100	NR	NR	NR	55	56.7 (14–87)	31:24	Prospective	Sciubba et al. classification	85 (85/100)	NR	NR	NR	8
Parker et al. [20]	2009	USA	161	NR	NR	NR	85	59.2 (18.1)	57:28	Retrospective	A breach was defined > 20% of screw outside of pedicle	93.1 (150/161)	NR	NR	NR	8
Yukawa et al. [21]	2009	Japan	23	NR	NR	NR	23 out of 620 screw of 144 patients	44.1 (14–90) of 144 patients	125:19	Retrospective	Yukawa et al. classification	65.2 (15/23)	NR	NR	NR	7
Payer et al. [22]	2009	Switzerland	NR	24	NR	NR	12	58 (23–78)	8:4	Prospective	Post-op CT, without classification	NR	91.7 (22/24)	NR	NR	5
De Iure et al. [23]	2009	Italy	20	NR	NR	NR	12	33.4 (14–62)	6:6	Retrospective	Post-op CT, without classification	100 (20/20)	NR	NR	NR	5
Simsek et al. [24]	2009	Turkey	34	NR	NR	NR	17	40 (6–74)	13:4	Retrospective	Post-op CT, without classification	100 (34/34)	NR	NR	NR	5
Tan et al. [25]	2009	China	22	NR	NR	NR	11 out of 17 patients	42.5 (25–67) of 17 patients	12:5	Retrospective	Post-op CT, without classification	100 (22/22)	NR	NR	NR	5
Xie et al. [26]	2009	China	50	NR	NR	NR	25	42 (18–70)	15:10	Retrospective	Post-op CT, without classification	100 (50/50)	NR	NR	NR	6
Miyamoto et al. [27]	2009	Japan	32	NR	NR	NR	32 out of 130 screw of 29 patients	61.2 (17.4)	19:10	Retrospective	Neo et al. classification	100 (32/32)	NR	NR	NR	7
Mueller et al. [28]	2010	Germany	47	NR	NR	NR	27	56 (22)	13:14	To 24-month postoperatively	Modified Gertzbein and Robbins	82.9	NR	NR	NR	8
Alosh et al. [29]	2010	USA	170	NR	NR	NR	93	57.9 (17.4)	59:34	Retrospective	Modified Gertzbein and Robbins	74.7 (127/170)	NR	NR	NR	8
Wang et al. [ 30]	2010	USA	638	NR	NR	NR	319	38.3 (4–73)	195:124	Retrospective	Wang et al. classification	92.8 (592/638)	NR	NR	NR	8
Lee et al. [30]	2010	South Korea	54	NR	NR	NR	27	51 (7–79)	11:16	Retrospective	Post-op CT, without classification	98.1 (53/54)	NR	NR	NR	6
Mummaneni et al. [31]	2010	USA	NR	76	NR	NR	38 out of 42 patients	64 (19–91)	24:18	Retrospective	Post-op CT, without classification	NR	100 (76/76)	NR	NR	6
Ni et al. [32]	2010	China	26	NR	NR	NR	13	48.5 (32–65)	9:4	Retrospective	Post-op CT, without classification	100 (26/26)	NR	NR	NR	5
Bransford et al. [33]	2011	USA	260	56	NR	NR	328	Over 7 years	188:140	Retrospective	Upendra et al. classification	98.8 (257/260)	94.6 (53/56)	NR	NR	9
Ishikawa et al. [34]	2011	Japan	NR	NR	24	NR	24 out of 108 screw of 21 patients	67.2 (42–83) of 21 patients	9:12	Retrospective	Neo et al. classification	NR	NR	Overall 88.9	NR	7
Hamilton et al. [35]	2011	USA	80	8	NR	NR	44	71 (67–89 )	23:21	Retrospective	Post-op CT, without classification	100 (80/80)	100 (8/8)	NR	NR	7
Chun et al. [36]	2011	South Korea	30	NR	NR	NR	15	56.8 (27–74 )	5:10	Retrospective	Post-op CT, without classification	100 (30/30)	NR	NR	NR	5
Nitising et al. [37]	2011	Thailand	NR	20	NR	NR	10	15–59	7:3	Retrospective	Post-op CT, without classification	NR	100 (20/20)	NR	NR	5
Lee et al. [38]	2011	South Korea	82	6	NR	NR	44	47.7 (4–84)	28:16	Retrospective	Post-op CT, without classification	95.1 (78/82)	100 (6/6)	NR	NR	7
Kang et al. [39]	2012	USA	NR	32	NR	NR	20	66 (19–89 )	9:11	Retrospective	Post-op CT, without classification	NR	96.9 (31/32)	NR	NR	5
Kawaguchi et al. [40]	2012	Japan	16	NR	NR	NR	16 out of 44 screw of 11 patients	57.4 (14–78 )	2:9	Retrospective	Neo et al. classification	100 (16/16)	NR	NR	NR	7
Ringel et al. [41]	2012	Germany	68	NR	NR	NR	35	64 (8–90)	20:15	Prospective	Post-op CT, without classification	82.3 (56/68)	NR	NR	NR	5
Jeon et al. [42]	2012	South Korea	28	6	NR	NR	17	40.4 (15–68)	9:8	Retrospective	Post-op CT, without classification	96.4 (27/28)	100 (6/6)	NR	NR	6
Tauchi et al. [43]	2013	Japan	NR	NR	37	NR	37 out of 196 screw of 46 patients	53.2 (5–84) of 46 patients	NR	Retrospective	Neo et al. classification	NR	NR	Overall 87.8	NR	6
Wu et al. [44]	2013	China	20	NR	NR	NR	10	45 (38–82)	6:4	Retrospective	Perforations of the pedicle wall (< 2 mm)	85 (17/20)	NR	NR	NR	7
Ling et al. [45]	2013	Singapore	20	NR	NR	NR	20 out of 103 screw of 21 patients	43 (6–83)	12:9	Retrospective	Neo et al. classification	90 (18/20)	NR	NR	NR	7
Yang et al. [46]	2013	China	24	NR	24	NR	24	45.9 (4.9)	11:13	Retrospective	Modified Neo et al. classification	95.8 (23/24)	NR	100 (24/24)	NR	9
Bydon et al. [47]	2014	USA	341	NR	NR	NR	181	57.9 (15.1)	101:80	Retrospective	Sciubba et al. classification	77.4 (264/341)	NR	NR	NR	8
Hojo et al. [48]	2014	Japan	148	NR	NR	NR	148 of 1065 screw of 283 patients	57.4 (14–87) out of 283 patients	183:100	Retrospective	Neo et al. classification	77.1 (114/148)	NR	NR	NR	8
Uehara et al. [49]	2014	Japan	NR	NR	33	NR	33 of 579 screw of 129 patients	63.4 (14.4) out of 129 patients	82:47	Retrospective	Uehara et al. classification	NR	NR	87.9 (29/33)	NR	8
Singh et al. [50]	2014	India	NR	NR	20	NR	10	17–81	9:1	Retrospective	Modified Gertzbein and Robbins classification	NR	NR	95 (19/20)	NR	7
Yu et al. [51]	2014	China	NR	NR	26	NR	26 of 108 screw of 23 patients	33.5 (19–52) of 23 patients	11:12	Retrospective	3D CT at the end of the procedure	NR	NR	96.1 (25/26)	NR	7
Tao et al. [52]	2014	China	NR	NR	64	6	70 out of 196 screw out of 99 patients	35 out of 99 patients	53:46	Retrospective	Modified Gertzbein and Robbins classification	NR	NR	89.1 (57/64)	100 (6/6)	9
Kim et al. [53]	2014	South Korea	NR	NR	32	NR	32 of 58 screw of 18 patients	45.8 (24–72)	13:5	Retrospective	Modified Neo et al. classification	NR	NR	84.3 (27/32)	NR	7
Kaneyama et al. [54]	2014	Japan	26	12	NR	NR	38 of 48 screw of 23 patients	69.4 (54–86)	10:13	Prospective	Neo et al. classification	100 (26/26)	100 (12/12)	NR	NR	8
Yang et al. [55]	2014	China	40	NR	NR	NR	20	40.2 (8–63)	11:9	Retrospective	Post-op CT, without classification	97.5 (39/40)	NR	NR	NR	6
Bredow et al. [56]	2015	Germany	NR	NR	65	NR	28	63.8 (16.8)	16:12	NR	Modified Gertzbein and Robbins classification	NR	NR	95.4	NR	8
Qi et al. [57]	2015	China	42	NR	NR	NR	21	46.5 (24–69)	13:8	Retrospective	Post-op CT, without classification	100 (42/42)	NR	NR	NR	6
Shih et al. [58]	2015	Taiwan	26	NR	NR	NR	13 of 35 patients	55.3 (21–7)	18:17	Retrospective	Post-op CT, without classification	96.1 (25/26)	NR	NR	NR	5
Lang et al. [59]	2016	China	NR	NR	40	NR	20	35.1 (18–55)	15:5	Retrospective	Gertzbein and Robbins classification	NR	NR	89.3% (50/56)	NR	8
Zheng et al. [60]	2016	China	172	NR	NR	NR	86	42.6 (16–69)	48:38	Retrospective	Post-op CT, without classification	100 (172/172)	NR	NR	NR	6
Zhao et al. [61]	2017	China	NR	NR	24	NR	12	37.4 (18–47)	12:0	Retrospective review of a prospectively collected data	3D CT at the end of the procedure	NR	NR	95.8 (23/24)	NR	7
Uehara et al. [62]	2017	Japan	NR	NR	40	NR	40 of 3413 screw of 359 patients	43 (26.9) of 359 patients	147:212 of 359 patients	Retrospective	Rao et al. classification	NR	NR	95 (38/40)	NR	8
Singh et al. [63]	2017	India	NR	NR	30	NR	15	34.4 (17–81)	13:2	Retrospective	Gertzbein and Robbins classification	NR	NR	93.3 (28/30)	NR	7
Shimokawa et al. [64]	2017	Japan	NR	NR	114	NR	114 of 762 screw of 128 patients	65.5 (15–92)	84:44 of 128 patients	Retrospective	Neo et al. classification	NR	NR	99.1 (113/114)	NR	8
Sugawara et al. [65]	2017	Japan	20	NR	NR	NR	20 of 48 screw of 12 patients	42–77	6:6	Prospective	3D/multiplanar imaging software	100 (20/20)	NR	NR	NR	7
Liu et al. [66]	2017	China	62	NR	NR	NR	31	51 (45–62)	18:13	Prospective	Post-op CT, without classification	100 (62/62)	NR	NR	NR	6
Jacobs et al. [67]	2017	Germany	NR	NR	60	NR	30	52 (3–91)	22:8	Retrospective	Gertzbein and Robbins classification	NR	NR	100 (60/60)	NR	8
Cao et al. [68]	2017	China	174	NR	NR	NR	87	39.2 (25–55)	NR	Retrospective	Modified Gertzbein and Robbins classification	95.9 (167/174)	NR	NR	NR	8
Guo et al. [70 ]	2017	China	25	NR	NR	NR	13	45.1 (25–57)	6:7	Prospective	Accuracy of the screw fixation was evaluated with the Mimics15.0 software	Overall 94.6	NR	NR	NR	6
Jiang et al. [71 ]	2017	China	108	NR	NR	NR	54	45.3 (12–54)	34:20	Prospective	Modified Gertzbein and Robbins	Overall 92.6	NR	NR	NR	7
Wu et al. [69]	2017	China	40	NR	NR	NR	20	NR	NR	Prospective	Accuracy of the screw fixation was evaluated with the Mimics software	100	NR	NR	NR	8
Pu et al. [70]	2018	China	34	NR	NR	NR	17	43.3 (25–56)	11:6	Retrospective	Kawaguchi et al. classification	Overall 97.06	NR	NR	NR	6
Pu et al. [71]	2018	China	98	NR	NR	NR	49	22–56	25:24	Retrospective	Kawaguchi et al. classification	Overall 86.5	NR	NR	NR	7
Sugawara et al. [72]	2018	Japan	138	NR	NR	NR	138 out of 813 screw of 103 patients	15–85	57:46	Prospective	3D/multiplanar imaging software	100 (138/138)	NR	NR	NR	8
Punyarat et al. [5]	2018	Thailand	52	87	NR	NR	76	59.9 (20–86)	42:34	Retrospective	Sciubba et al. classification	76.9 (40/52)	88.5 (77/87)	NR	NR	9
Pham et al. [73]	2018	USA	40	NR	NR	NR	24	56.1 (23–91)	18:6	Retrospective	Sciubba et al. classification	82.5 (33/40)	NR	NR	NR	8
Ould-Slimane et al. [74]	2018	France	NR	NR	22	NR	11	55 (22–69)	6:5	Prospective	No cortical breach was detected using cone-beam CT at the end of the procedure	NR	NR	100	NR	5
Chachan et al. [75]	2018	Singapore	NR	NR	32	NR	32 of 241 screw of 44 patients	62.1 (34–81)	27:17	Retrospective	Gertzbein and Robbins classification	NR	NR	100	NR	7
Marco et al. [76]	2018	USA	29	NR	NR	NR	22 of 30 patients	54 (6–87)	15:15	Retrospective	One cortical breach, which measured less than 2 mm, was detected.	96.5 (28/29)	NR	NR	NR	5
Sai Kiran et al. [77]	2018	India	24	49	NR	NR	94	30 (16.3)	61:33	Retrospective	Upendra et al. classification	100 (24/24)	100 (49/49)	NR	NR	9
Işik et al .[78]	2018	Turkey	24	8	NR	NR	16 of 28 of patients	44.7 (21–73 )	11:17	Retrospective	Post-op CT, without classification	100 (24/24)	100 (8/8)	NR	NR	6
Park et al. [79]	2019	South Korea	NR	76	NR	NR	58	62.4 (14.5)	20:38	Retrospective	Modified Upendra	NR	97.4	NR	NR	8
Zhang et al. [80]	2019	China	68	NR	NR	NR	36	6.9 (3.2)	21:15	Retrospective	Smith classification	98.5 (67/68)	NR	NR	NR	8
Wu et al. [9]	2019	China	NR	NR	54	NR	27	38.5 (22–62)	17:10	Prospective	3D model simulation software	NR	NR	100 (54/54)	NR	8
Tian et al. [81]	2019	China	52	12	50	14	64	46.4 (10.7)	40:24	Retrospective	Hlubek et al. classification	96.15 (50/52)	91.67 (11/12)	84 (42/50)	85.7 (12/14)	8
Hur et al. [82]	2019	South Korea	NR	NR	92	NR	48	58.8 (35–80)	30:18	Retrospective	Gertzbein and Robbins	NR	NR	91.3 (82/92)	NR	8
Carl et al. [83]	2019	Germany	NR	NR	26	NR	16	72.7 (24–84)	7:9	Retrospective	Laine et al. classification	NR	NR	96.2 (25/26)	NR	7
Lee et al. [84]	2020	South Korea	26	1	32	1	34 (15 F:19 N)	54.8 (16.7)	18:16	Retrospective	Gertzbein and Robbins	88.5	NR	93.8	NR	9

*NR* not reported

### Assessing screw placement accuracy

The accuracy of C2 pedicle/pars screws placement was determined with intraoperative/postoperative CT imaging. There are 12 reported types of classification for assessing accuracy of C2 screw placement. Most studies used the Gertzbein et al. classification [88]. A summary of classifications and studies that used them is provided in Table 3 [7, 19, 21, 40, 49, 79, 85, 88–93].

**Table 3:** Accuracy rate classifications for screw insertion

Name of classification	Year	Description	Studies used the classification
Gertzbein and Robbins [88]	1990	Grade 0, when a screw was placed inside the bone; grade I, screw perforation of the cortex within 2 mm; grade II, screw perforation from 2 to 4 mm; and grade III, screw perforation of more than 4 mm. In some of articles, this classification was modified [28, 56]. Grade 0 is considered the accuracy of in C2 screw placement [28].	[17, 28, 29, 50, 52, 56, 59, 63, 67, 68, 75, 82, 84, 87]
Laine et al. [89]	2000	Based on CT images, in this classification, screw position was staged as screw inside the pedicle or perforation of the pedicle cortex by up to 2 mm, from 2 to 4 mm, from 4 to 6 mm, or by more than 6 mm. Type I and type II were categorized as acceptable placement.	[83]
Rao et al. [90]	2002	Each screw position was assigned a grade from 0 to 3, as follows: grade 0 reflected no perforation of the pedicle; grade 1 indicated less than 2 mm of perforation of the pedicle; grade 2 represented 2–4 mm of perforation of the pedicle; and grade 3 reflected perforation greater than 4 mm. Grades 2 and 3 insertions were judged to be major perforations. Overall, it is considered a perforation of less than 2 mm to be satisfactory.	[62]
Neo et al. [91]	2005	Screw positions were classified into four grades: grade 0, no perforation, and the screw was completely contained in the pedicle; grade 1, perforation < 2 mm (that is, less than half of the screw diameter); grade 2, perforations ≥ 2 mm but < 4 mm; and grade 3, perforation ≥ 4 mm(complete perforation). The screw was classified as grade 0 be acceptable.	[27, 34, 40, 43, 45, 46, 48, 53, 54]
Upendra et al. [92]. It was modified by Park et al. [79]	2008	Type I, ideal placement—screw threaded completely within bony cortex; type IIa, acceptable placement—< 50% of the diameter of the screw violating surrounding cortex and screw protrusion of < 1 mm from the anterior cortex for pedicle and pars screws; type IIb, relatively acceptable placement—screw violating < 33% of the diameter of the C2 transverse foramen (TF); type IIc, relatively unacceptable placement—screw violating ≥ 33% of the diameter of the C2 TF or ≥ 50% of diameter of screw violating surrounding cortex; type III, unacceptable placement—clear violation of TF or spinal canal; regardless of clinical neurovascular complications. Overall, types I, IIa, and IIb were categorized as acceptable placement and types IIc and III as unacceptable placement.	[33, 77, 79]
Sciubba et al. [19]	2009	It is described by location (lateral, medial, inferior, and superior) and percentage of screw diameter over cortical edge (0 = none; grade I = < 25% of screw diameter; grade II = 26–50%; grade III = 51–75%; and grade IV = 76–100%). Type 0 was categorized as acceptable placement.	[5, 19, 47, 73]
Yukawa et al. [21]	2009	The accuracy of the placement of the pedicle screws into the medial/lateral pedicle walls was evaluated on axial CT scans (2 mm slices), whereas superior/inferior pedicle wall screw location was examined on oblique radiographs. Incorrect screw placement was classified as either screw exposure or pedicle perforation. A screw was exposed if it broke the pedicle wall, but more than 50% of the screw diameter remained within the pedicle. A pedicle perforation occurred if a screw breached the pedicle wall, and more than 50% of the screw diameter was outside the pedicle.	[21]
Wang et al. [85]	2010	This classification was based on axial plane, para-sagittal plane, and coronal plane. The grading has been described elsewhere in detail [85].	[85]
Kawaguchi et al. [40]	2012	Grade 0, the screw was completely located in the vertebral pedicle; grade I, the screw penetrated the pedicle bone cortex < 2 mm without complications; grade II, the screw penetrated the pedicle bone cortex > 2 mm without complications; and grade III, complications related to screw placement occurred, such as nerve and vertebral artery injuries. Grade 0 was considered to be the correct location of pedicle screws and safe placement.	[70, 71]
Uehara et al. [49].	2014	The screw insertion status was classified as grade 1 (no perforation), indicating that the screw was accurately inserted in pedicle; grade 2 (minor perforation), indicating perforation of less than 50% of the screw diameter; and grade 3 (major perforation), indicating perforation of 50% or more of the screw diameter. The screw was classified as grade 1 be acceptable.	[49, 62]
Smith et al. [93]	2016	On postoperative CT scans, type I was defined as ideal placement without cortical violation; type II was an acceptable placement with less than half the diameter of the screw violating the surrounding cortex and less than 1 mm protrusion from the anterior cortex; and type III is an unacceptable placement with clear violation of the transverse foramen or spinal canal.	[80]
Hlubek et al. [7]	2018	Grade A, screw completely confined within cortical surfaces; grade B, transverse foramen violation with the screw obstructing 1–25% of the foramen; grade C, transverse foramen violation with the screw obstructing 26–50% of the foramen; grade D, transverse foramen violation with the screw obstructing 51–75% of the foramen; grade E, transverse foramen violation with the screw obstructing 76–100% of the foramen; grade M, medial breach into the spinal canal. Grades A and B were determined to be acceptable placement, and Grades C–E and M were determined to be unacceptable.	[81]

### Study characteristics and quality assessment

The characteristics of each study are shown in Table 2. Fifty-seven studies were conducted in Asian countries, 12 studies in North America, and 10 studies in Europe. Sixty-seven studies were retrospective, and 12 were prospective in design. Sample size ranged from 10 to 328 patients. The reported accuracy rate ranged from 65.2 to 100% for patients after cervical surgery. The NOS for each study can be found in Table 2. All of the studies analyzed in this systematic review scored five or above, which is considered of moderate to high quality studies [10], and 52 of the studies were considered high-quality studies.

### Meta-analysis

**Fig. 2:**
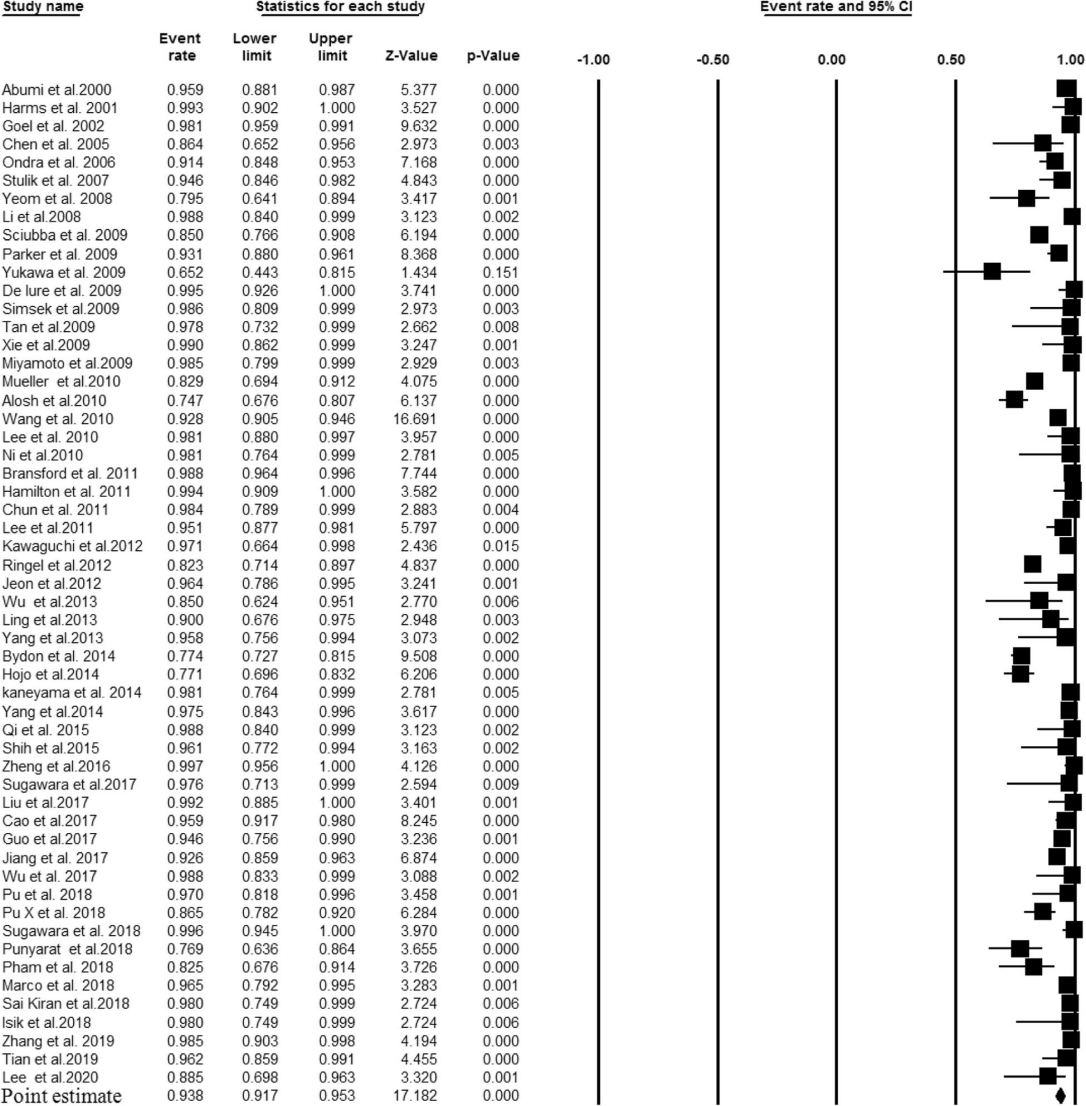
Point estimates with 95% confidence intervals and forest plot of studies reporting on accuracy rates of fusion following posterior atlantoaxial fusions with C2 pedicle screw and free-hand technique

**Fig. 3:**
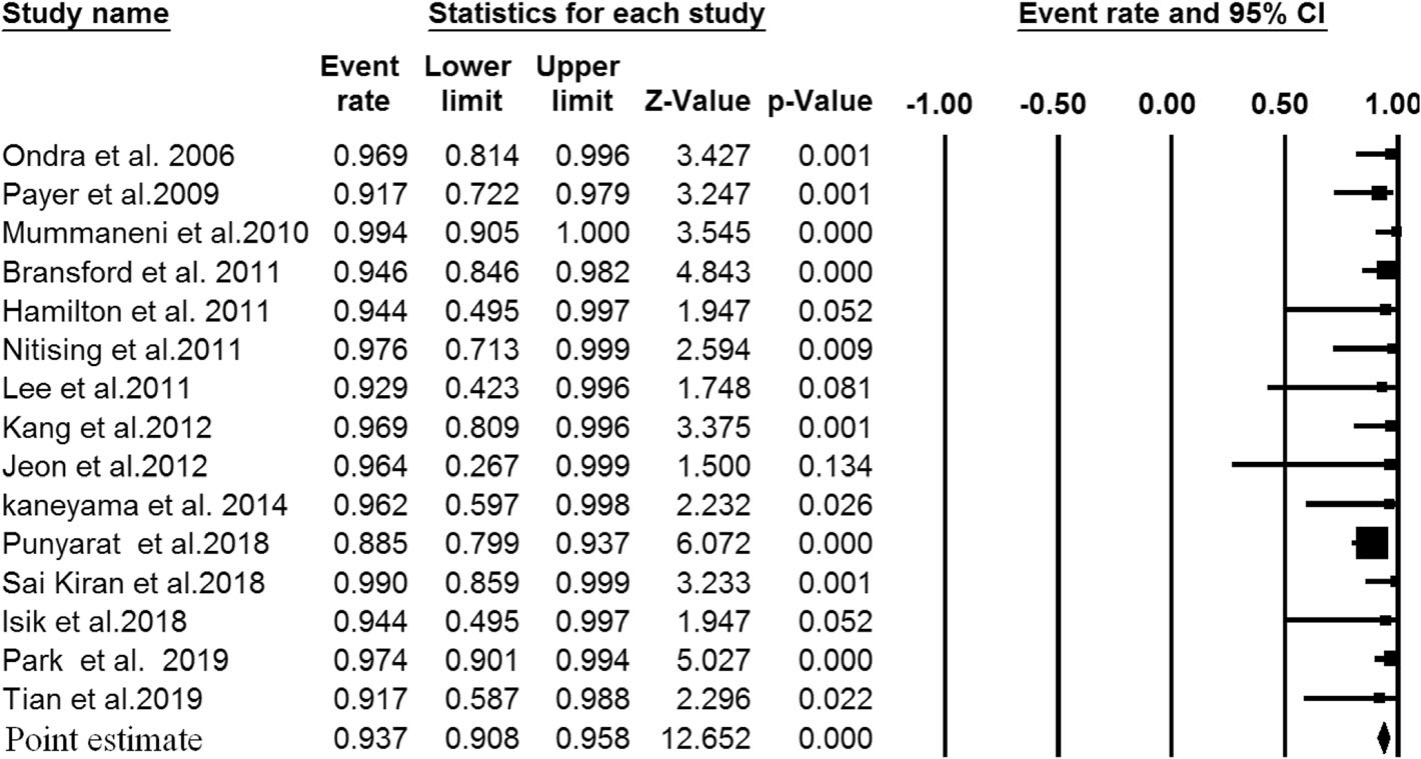
Point estimates with 95% confidence intervals and forest plot of studies reporting on accuracy rates of fusion following posterior atlantoaxial fusions with C2 pars screw and free-hand technique

**Fig. 4:**
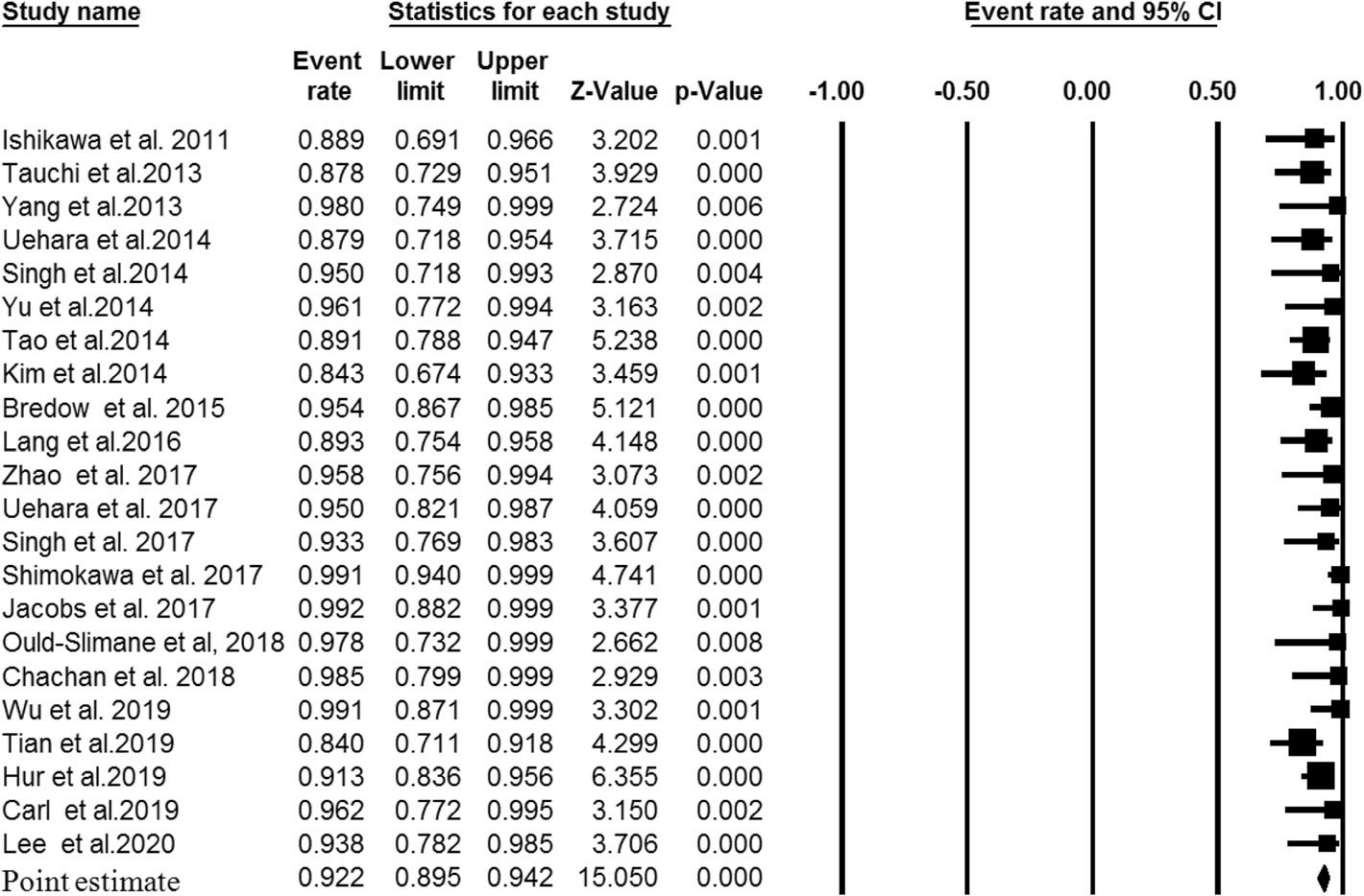
Point estimates with 95% confidence intervals and forest plot of studies reporting on accuracy rates of fusion following posterior atlantoaxial fusions with C2 pedicle screw and navigation technique

**Fig. 5:**
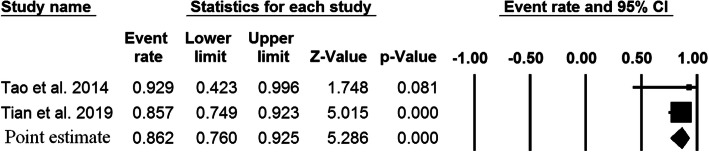
Point estimates with 95% confidence intervals and forest plot of studies reporting on accuracy rates of fusion following posterior atlantoaxial fusions with C2 pars screw and navigation technique

A total of 79 studies, comprising 4431 patients with upper cervical fusion, were included in the meta-analysis. Overall, 6026 C2 pedicel/pars were used as follows: C2 pedicle free-hand (*n* = 4558), C2 pars free-hand (*n* = 506), C2 pedicle navigation (*n* = 941), and C2 pars navigation (*n* = 21). There were 55 studies indicating the association between the pedicle screw placement and the accuracy rate of upper cervical fusion patients. Since there was significant heterogeneity among the above 55 studies (I-squared value = 79.8% and *P* value < 0.001), we performed a random effects model to assess the pooled accuracy rate estimate and corresponding 95% CI. As shown in Fig. 2, the accuracy rate of the C2 pedicle screw free-hand technique was 93.8% (*P* value < 0.001). Forest plot for C2 pars screw placement of free-hand technique (15 studies, I-squared value = 0.0%, and *P* value = 0.599), C2 pedicle screw placement of navigation technique (22 studies, I-squared value = 21.63%, and *P* value = 0.178 ), and C2 pars screw placement of navigation technique (2 studies, I-squared value = 0.0%, and *P* value = 0.608 ) are shown in Fig. 3 (a fixed effects model; accuracy rate 93.7%; *P* value < 0.001), Fig. 4 (a fixed effects model; accuracy rate 92.2%; *P* value < 0.001 ), and Fig. 5 (accuracy rate 86.2%; *P* value < 0.001), respectively. In this systematic review study, no statistically significant results were observed between the accuracy of placement C2 pedicle versus C2 pars in free-hand technique and the free-hand C2 pedicle group versus the navigated C2 pedicle group (all, *P* value > 0.05).

### Publication bias

Publication bias was measured by Begg’s test. For C2 pedicle screw of free-hand technique, the *P* value for Begg’s test was 0.117, indicating that there was no significant publication bias among the included studies. Also, the *P* value for Begg’s test was 0.766 for the C2 pars screw free-hand technique. Funnel plot and Begg’s test did indicate obvious published bias for C2 pedicle screw of navigation technique (*P* = 0.001). In addition, due to studies, less than 3 Begg’s test was not performed for C2 pedicle screw of navigation technique.

## Discussion

To our knowledge, no previous systematic review, with or without meta-analysis, has been reported with the same purpose and methods. The analysis of the literature reveals that there are many studies fulfilling the inclusion criteria of the present systematic review. That is why the current study can include 79 studies. Statistical analyses showed that the placement accuracy rate for the free-hand C2 pedicle group was comparable to that for the navigated C2 pedicle group and between C2 pedicle and pars screws placement. Overall, the free-hand technique was not found to accurate than navigation for C2 pedicle/pars screw placement.

In this study, there was no difference in the safety and accuracy between the free-hand and navigated techniques, which could be for the following reasons: (a) Screw guide template studies with the highest precision and accuracy were considered free-hand technique. (b) Experience with navigation system also plays a role in this arena. (c) Less number of navigation system studies compared to free-hand technique due to the lack of popular accessibility and (d) heterogeneity in studies.

### Study consistency

Of the 79 articles, only 12 fully reported on patients’ recruitment or the source of prospective data. No randomized trial was found. Learning curve and size of screws were not consistently reported, resulting in a potential bias. The surgical approach was described in nearly all studies, while new entry point and trajectory, which could indicate a potential for screw malposition, were not consistently reported. For accuracy assessment of C2 pars/pedicle screw placement, a variety of grading criterion are reported in the literature. Comparison between accuracy rates was limited by the presence of twelve different definitions of accuracy rate and twenty-five studies (31.6%; 25/79) not presenting any definition. In addition, 14 articles (17.7%; 14/79) used the Gertzbein and Robbins grading system for evaluation of accuracy of screw placement. In a review study of C2 pedicle screw placement, Elliott et al. [94] showed that the incidence of malposition, confirmed by CT scan, varied from 1.1 to 44% in cases with fluoroscopic guidance. However, in this systematic review, the reported accuracy rate ranged from 65.2 to 100%. This wide range could be a result of varying classification method of screw displacement among studies.

### Study quality

Only 59.4% (47/79) of studies used a clearly defined accuracy rate classification definition. Most studies were small with an average study group size of 44 patients dropping to 31 when removing the eight studies with over 100 patients. The method of screw insertion was well defined, or a pre-defined method was cited. In some of studies, the type and size of screws was not specified. Only two studies [52, 84] assessed the accuracy rate of navigated C2 pars screw malposition, and data were limited for comparison. Therefore, further research with large sample sizes comparing accuracy rates of navigation with free-hand methods is warranted.

Studies included heterogeneous populations with varying pathological types. However, accuracy of either procedure should not have been affected by pathology. Furthermore, more complex pathology or anatomy was not reason for choosing navigation over free-hand technique or vice versa [7]. Also, here was considerable regarding the length of C2 pars/pedicle screw, navigated technique, surgeon’s experience, and grading criteria of accuracy, which can affect results. A standardized assessment process, moving forward, would greatly assist in future analyses in this arena. According to this 20-year study (2000–2020), over the past 20 years, numerous navigation systems such as MR-based navigation, CT-guided navigation, and O-arm-based navigation have been developed. Each of these systems has strengths and weaknesses concerning yield, cost, speed, and learning carve. Hence, it may cause heterogeneity to put all navigation systems in the same group. Albeit, it could be evaluated separately in the future.

Until now, a few studies have compared the accuracy of C2 pedicle and pars screw placement for atlantoaxial fusion [7, 84]. Lee et al. showed that O-arm navigation slightly improved the accuracy rate of C2 pedicle screw positioning, compared to the free-hand technique, though statistically meaningful results were not reported [84]. A C2 screw accuracy rate was reported to be 100% by Wu et al. [9]. They used 3D model simulation software for better evaluation of anatomy and then applied this to the navigation process [9]. Contrary to their study, Hlubek et al. found that the free-hand technique was significantly more accurate than CT-based navigation for C2 pedicle/pars screw placement [7]. Hence, illustrating the ongoing challenge associated with data analysis.

The corridor for C2 pedicle and pars screw placement is often narrow. Hence, it would seem that navigation techniques would present a natural solution to this corridor definition challenge in anatomically complex cases. There are several advantages of using an intraoperative image guidance for cervical surgery, including multi planar CT images of different operative levels in a single sequence can be achieved to increase accuracy of surgery, decreased radiation exposure to the surgeon and patient, and screw positions can be tested in the surgical field, which will reduce the failure rates [84]. On the other hand, surgical landmarks and fluoroscopy have been applied routinely for pedicle screw insertion, but a number of studies disclose inaccuracies in placement using these conventional techniques. Moreover, the free-hand technique is safe and accurate when it is in the hands of an experienced surgeon [95]. Then, it could be argued that the use of the navigation for C2 pars and pedicle placement is better than free-hand technique. However, there are many probable sources of error with the navigated method that resulted in less accurate screw placement. The CT image may be distorted because of metal artifacts from prior implant placement and the extra time required to set up the navigation system [84]. Also, the motion of C2 relative to the reference frame may introduce error. In addition, registration inaccuracies could be related to lack of correspondence between the pre-operative CT image, obtained in the standard supine position, and the intraoperative prone position, especially in patients with cervical instability. Other sources of inaccuracies include accidental displacement or reference frames [7]. Hence, in order to correct the source of error, further research is required to provide evidence of the precise cause of inaccuracy with navigated C2 pedicle and pars screw placement.

### Strengths and limitations

The strengths of this review include the broad search strategy in four major databases and high sensitivity of the abstract search. This study has several limitations, though. First, this is a meta-analysis carried out at study level, meaning that different confounding factors from the patient level were not evaluated and included in the analysis. Second, the search was limited to English publications. Potentially relevant studies could have been missed. Third, although it seems that the CT-based navigation could be useful in C2 pedicle screw placement, this intraoperative CT navigation is not universally available. Moreover, it is mandatory to consider the radiation exposure for operative staff, which is significantly higher with CT-based navigated than with standard techniques. Fourth, all studies were performed retrospectively. To the best of our knowledge, no prior prospective randomized control studies have been performed to compare the safety and accuracy of the free-hand technique versus navigation for the placement of C2 pedicle and pars screws; hence, a high level of evidence was lacking in our review. Finally, the main limitation of the study was the high level of heterogeneity in the methods used among the included trials. In particular, there were heterogeneities in (1) variety in surgical technique and screw guide templates, (2) variety in navigation systems, (3) the screw placement accuracy measures applied, (4) length and size of screw (presently, there are no criteria on the size of C2 pedicle screws that maximizes the C2 accuracy rate placement), (5) the learning curve associated with using free-hand techniques and navigation systems, (6) costs from acquiring guidance technology, and (7) radiation exposure. These items were not discussed in the included articles, but it would be of interest in future prospective studies.

## Conclusion

The C2 pedicle/pars placement accuracy rate for the free-hand group was comparable to that for the navigated group. Further randomized controlled trials with large sample sizes comparing accuracy rates of navigated with free-hand methods are warranted to complement the existing evidence.

## Data Availability

Data sharing not applicable to this article as no datasets were generated. All datasets reviewed in this article are cited in the results section.

## Abbreviations

NOS: Newcastle-Ottawa Scale
CMA: Comprehensive Meta-Analysis Software
